# Are Physical activity and Benefits Maintained After Long-Term Telerehabilitation in COPD?

**DOI:** 10.5195/ijt.2016.6200

**Published:** 2016-12-15

**Authors:** HANNE HOAAS, BENTE MORSETH, ANNE E. HOLLAND, PAOLO ZANABONI

**Affiliations:** 1 NORWEGIAN CENTRE FOR E-HEALTH RESEARCH, UNIVERSITY HOSPITAL OF NORTH NORWAY, TROMSØ, NORWAY; 2 FACULTY OF HEALTH SCIENCES, UIT THE ARCTIC UNIVERSITY OF NORWAY, TROMSØ, NORWAY; 3 DEPARTMENT OF COMMUNITY MEDICINE, UIT THE ARCTIC UNIVERSITY OF NORWAY, TROMSØ, NORWAY; 4 SCHOOL OF SPORTS SCIENCE, UIT THE ARCTIC UNIVERSITY OF NORWAY, TROMSØ, NORWAY; 5 LA TROBE UNIVERSITY, MELBOURNE, AUSTRALIA; 6 ALFRED HEALTH, MELBOURNE, AUSTRALIA; 7 INSTITUTE FOR BREATHING AND SLEEP, MELBOURNE, AUSTRALIA

**Keywords:** COPD, Maintenance, Physical activity, Pulmonary rehabilitation, Quality of life, Telemedicine, Telerehabilitation

## Abstract

This study investigated whether physical activity levels and other outcomes were maintained at 1-year from completion of a 2-year telerehabilitation intervention in COPD. During the post-intervention year, nine patients with COPD (FEV_1_ % of pred. 42.4±19.8%; age 58.1±6 years) were encouraged to exercise on a treadmill at home and monitor daily symptoms and training sessions on a webpage as during the intervention. Participants were not provided supervision or motivational support. Physical activity levels decreased from 3,806 steps/day to 2,817 steps/day (p= 0.039). There was a decline in time spent on light physical activity (p=0.009), but not on moderate-to-vigorous activity (p=0.053). Adherence to registration of symptoms and training sessions decreased significantly. Other outcomes including health status, quality of life, anxiety and depression, self-efficacy, and healthcare utilization did not change significantly. In conclusion, provision of equipment for self-management and unsupervised home exercise might not be enough to maintain physical activity levels.

Patients with Chronic Obstructive Pulmonary Disease (COPD) suffer from a persistent airflow limitation which, together with commonly experienced extrapulmonary effects and comorbidities, is associated with physical inactivity (Global Initiative for Chronic Obstructive Lung Disease (GOLD), 2016; Suissa, Dell’Aniello, & Ernst, 2012; Watz et al., 2014). An inactive lifestyle might in turn lead to a more rapid progression of the disease and worsening of health status (Troosters et al., 2013). Inactivity is also associated with a higher risk of COPD-related hospital admissions (Garcia-Aymerich, Lange, Benet, Schnohr, & Antó, 2006). Pulmonary rehabilitation (PR) is highly recommended for all patients with COPD, and is effective in reducing dyspnea, improving functional exercise capacity, and quality of life (Global Initiative for Chronic Obstructive Lung Disease (GOLD), 2016; Spruit et al., 2013). However, PR shows only modest effects in enhancing daily physical activity in patients with COPD (Burtin et al., 2015; Watz et al., 2014). Benefits of PR seem to diminish after 6–12 months without any maintenance strategy. Low adherence to long-term regular exercise might be one of the main reasons (Spruit et al., 2013). The optimal maintenance intervention to extend benefits of PR and enhance or prevent loss of physical activity over the long-term still remains unknown (Brooks, Krip, Mangovski-Alzamora, & Goldstein, 2002; Ries, Kaplan, Myers, & Prewitt, 2003; Spruit & Singh, 2013; Watz et al., 2014). However, continuity in follow-up programs and access to regular support and supervision is reported to support long-term exercise maintenance in COPD (Hellem, Bruusgaard, & Bergland, 2012). Telemedicine-based interventions promoted through phone calls, websites, or mobile phones have showed promising results in increasing physical activity levels in COPD (Lundell, Holmner, Rehn, Nyberg, & Wadell, 2015). Nevertheless, few studies have been conducted on the effects of telerehabilitation in COPD, and there is little knowledge about the long-term effects. Eight weeks of telerehabilitation comprising bicycle exercise remotely supervised via videoconferencing and self-management education provided on a computer resulted in significant improvements in exercise tolerance and quality of life (Marquis, Larivée, Dubois, & Tousignant, 2014). However, participants did not keep the exercise equipment after the intervention ended and improvements were not maintained after 6 months.

We conducted a pilot study on a 2-year telerehabilitation intervention for patients with COPD (Zanaboni, Lien, Hjalmarsen, & Wootton, 2013). This study demonstrated positive outcomes in terms of exercise maintenance, functional walking capacity, health status, and quality of life (Zanaboni, Hoaas, Lien, Hjalmarsen, & Wootton, 2016). Importantly, factors affecting satisfaction with the intervention from the participants’ view included experienced health benefits, increased self-efficacy and independence, emotional safety due to regular meetings, and access to specialist competence. Maintenance of motivation was a challenge and might have affected long-term adherence to the intervention (Hoaas, Andreassen, Lien, Hjalmarsen, & Zanaboni, 2016). After completing the study, participants kept the equipment and were reassessed at the end of the post-intervention year. Our hypothesis was that adherence to self-management and home exercise routines would decline without supervision and that patients therefore would not maintain physical activity and patient-reported outcomes like health status, quality of life, self-efficacy, and anxiety and depression in the post-intervention year, with more frequent COPD-related hospitalizations.

## METHODS

### STUDY DESCRIPTION AND INTERVENTION

A 2-year pilot study was conducted to test the benefits of a long-term telerehabilitation intervention for patients with COPD (Zanaboni et al., 2016). Equipment included a treadmill, a pulse oximeter, and a tablet computer. Participants were recommended to perform home-based exercise three times per week. They also had access to a webpage for self-management, which included an individually tailored interval training program, a daily diary for reporting symptoms and oxygen saturation at rest, and a training diary for reporting exercise duration, perceived exertion, oxygen saturation, and heart rate during exercise. A physiotherapist supervised the participants remotely via videoconferencing on a weekly basis. Details on the intervention and the exercise program are described elsewhere (Zanaboni et al., 2016).

At the end of the 2-year intervention period, participants kept the equipment and were encouraged to continue performing home-based exercise on the treadmill on their own and to record daily symptoms and training sessions on the webpage. However, during the post-intervention year they no longer received any supervision, motivational, or self-management support from the physiotherapist via videoconferencing. That is, they only kept the tools to perform unsupervised exercise at home and self-management.

The primary outcome of this study was change in physical activity level. Secondary outcomes included adherence to daily registrations and training sessions, health status, quality of life, self-efficacy, anxiety and depression, subjective impression of change, and healthcare resource utilization. We also wanted to explore motivational factors affecting maintenance to unsupervised home exercise.

A one-group pretest-posttest design was used. Data were collected both along the 2-year intervention period (at baseline (T0), one year (T1), two years (T2)), and at the end of the post-intervention year (T3). The current study focuses on comparing the post-intervention year (T3) with the last intervention year (T2). The study was approved by the Regional Committee for Medical and Health Research Ethics, North Norway. All participants gave written informed consent.

### DATA COLLECTION AND ANALYSIS

Baseline patients’ characteristics in terms of age, gender, body mass index [BMI], lung function (forced expiratory volume in 1 second as percentage of predicted values [FEV_1_ (% of pred.]), functional walking capacity (six-minute walk distance [6MWD]), and residential status were collected at T2 only. All study outcomes were collected at T2 (May 2014) and T3 (May 2015). Data at T2 were collected during an in-clinic visit, whilst data at T3 were collected by mail correspondence. All study objectives and time points for data collection are given in Table 1.

Physical activity levels were objectively measured by an activity monitor validated for people with COPD (Bodymedia SenseWear Armband) at T2 and T3 (Patel, Benzo, Slivka, & Sciurba, 2007). Participants were asked to wear the activity monitor for 7 consecutive days, day and night, except for a 1-hour break daily, normally for personal hygiene. The minute-by-minute output from the activity monitor was exported for further analysis using SenseWear Professional, version 8.0 software. The first and the last day of recording were excluded from analysis due to incomplete measurements which could introduce a bias. Hence, the minimum required measurement period was 5 days with wear time ≥10 hours, including at least one weekend day. Measurements for four weekdays of ≥8–10 hours wear time during waking hours are considered sufficient to obtain reliable data on physical activity in patients with COPD (Byrom & Rowe, 2016; Demeyer et al., 2014). Physical activity outcomes were measured energy expenditure in kilojoules (kJ) per day, total number of steps per day, minutes of awake sedentary time per day, total minutes of light physical activity per day (LIPA), total minutes of moderate-to-vigorous activity per day (MVPA), and total minutes in ≥10 minutes bouts of moderate-to-vigorous activity (MVPA bouts). Awake sedentary time is here defined as activity where the metabolic equivalent is between 0<1.5 METs, and measured sleep, as registered by the SenseWear armband, is excluded (Byrom & Rowe, 2016). Non-wear time is not included in measurement of sedentary behavior. By definition, LIPA refers to any activity between 1.5<3 METs (Norton, Norton, & Sadgrove, 2010). Any activity ≥3 METs was considered MVPA (Norton et al., 2010).

Use of the equipment and adherence were measured through the analysis of logs on the webpage during the last year of the telerehabilitation intervention and the post-intervention year. The average number of diary registrations per week and the average number of training sessions per week were computed for each participant. The adherence rate was calculated according to the recommendation of regular daily diary registrations and three training sessions per week, which were the same as in the telerehabilitation intervention. Details reported in the commentary field of the diaries during the intervention and the post-intervention year were also analyzed. In addition, use of the equipment and the webpage was assessed with a 5-point categorical scale (never, seldom, monthly, weekly and daily).

Patient-reported outcomes collected at T2 and T3 included health status and health-related quality of life, measured through the COPD Assessment Test [CAT] and the EQ-5D questionnaire, respectively. The minimal important difference (MID) for CAT is estimated as two points (Kon et al., 2014). Assessment of self-efficacy along with anxiety and depression were measured through the General Self-Efficacy Scale [GSES] and the Hospital Anxiety and Depression Scale [HADS], respectively. In addition, subjective impression of change was expressed by the Patients’ Global Impression of Change [PGIC]. A change-score ≥5 is considered a favorable change in their condition, including activity, limitations, symptoms, emotions, and overall quality of life (Hurst & Bolton, 2004). Moreover, a 2-point change from the last reported score is considered clinically significant (Hurst & Bolton, 2004).

The study also investigated changes in healthcare resource utilization between the last year of the telerehabilitation intervention (T2) and the post-intervention year (T3). Data on hospitalizations and outpatient visits were collected from the Norwegian Patient Registry. Costs were calculated from the diagnosis-related group (DRG) points specifically attributed to hospitalizations and outpatient visits and the public tariff per DRG point offered by the healthcare authority.

Motivational factors for maintaining exercise during the post-intervention year were explored using open-ended questionnaires at T3. The questionnaire consisted of three questions: (i) If you have missed the follow-up by telerehabilitation, which specific features have you missed? (ii) If you have exercised on your own, what has motivated you to continue exercising? (iii) Do you have anything else to tell us? A thematic analysis technique was used to identify topics in the written material (Braun & Clarke, 2006). One author structured the material, and three authors analyzed it individually before a mutual consensus was agreed upon.

### STATISTICAL ANALYSES

Descriptive data were reported as mean and standard deviation for continuous variables, and counts for categorical variables. Normality of distribution was tested by the Shapiro-Wilk Test. Subjects with missing data on one outcome measure were excluded from the analysis for that particular outcome measure. Multiple imputation was not performed due to the small sample. Changes in physical activity and other outcomes from the second year of telerehabilitation to the post-intervention year were tested with the paired-samples t-test. Results were reported as mean, standard deviation and mean difference with 95% confidence interval (CI). A value of p<0.05 was considered statistically significant. All statistical analyses were performed with IBM SPSS Statistics Version 22.

## RESULTS

### STUDY POPULATION

Nine patients with moderate to severe COPD who participated in long-term telerehabilitation were followed-up during the post-intervention year. Patients’ characteristics at T2 are shown in Table 2.

### PHYSICAL ACTIVITY

Physical activity data were available for 7 out of 9 participants. One participant had missing data from T2 due to an ongoing and prolonged exacerbation at the end of the telerehabilitation intervention. Another participant had missing data at T3 due to an allergic reaction to the armband experienced at the end of the post-intervention year. On average, participants had valid measurements from six days with recording of ≥22 hours on both occasions.

One year post intervention, average energy expenditure decreased from 9451 kJ per day to 8270 kJ per day (p=0.011, Table 3). Physical activity decreased from an average of 3806 steps/day to 2817 steps/day. This decline between time points was statistically significant (p=0.039). On average, the time spent in awake sedentary activity differed with one minute between time points (p=0.973). There was a 21% decline in time spent in LIPA (p=0.009). On average, total time spent in MVPA dropped by 51% between time points, from 88±66 minutes per day to 43±34 minutes per day, although this was not statistically significant (p=0.053). Time spent in MVPA bouts decreased by 53% (p=0.110). At the end of the intervention, 6 of the 7 participants had ≥150 weekly minutes of MVPA bouts, whereas only two participants maintained that level one year after the intervention.

### ADHERENCE AND USE OF THE EQUIPMENT

Adherence to registration of daily symptoms decreased from 39.3% during the second year of telerehabilitation to 15.6% during the post-intervention year (Table 4). Adherence to registration of home-based training sessions decreased from 42.9% to 13.9%. There was a statistically significant reduction in the use of both the daily diary (p=0.010) and training sessions (p=0.004). Only two of the participants actively used the webpage during the post-intervention year, with adherence to registration of daily symptoms and training sessions of 68.7% and 58.8%, respectively. During the intervention year, these two participants had an adherence rate of 87.2% to registrations in the daily diary and 98.8% for the training sessions. Both participants started reporting other forms of exercise and physical activity than home-based treadmill training (e.g. stationary bicycling, outdoor walking, snow shoveling, lawn mowing) in the post-intervention year. For these activities, they reported perceived exertion, lowest oxygen saturation, highest heart frequency, and duration as for treadmill training. One of the participants kept reporting in the daily diary detailed comments regarding everyday life, travels, sickness, and use of medication for 1.5 years after the end of the intervention.

On the questionnaire regarding use of the equipment, six participants answered they had used the treadmill and the pulse oximeter monthly or more. Four also reported using the webpage monthly or more.

### HEALTH STATUS AND QUALITY OF LIFE

There was no clinically or statistically significant change in the CAT score at the end of the post-intervention year (p=0.388, Table 5). The utility score calculated from the EQ-5D and the EQ Visual Analogue Scale (EQ VAS) were also not statistically significant different (p=0.070 and p=0.718, respectively).

### ANXIETY AND DEPRESSION

Data on anxiety and depression were available for all 9 participants. There was no statistically significant change in the average score for anxiety and depression measured with the HADS (Table 5).

### SELF-EFFICACY AND SUBJECTIVE IMPRESSION OF CHANGE

There was no difference in scores for self-efficacy and subjective impression of change between the end of the second intervention year and the end of the post-intervention year (Table 5). At the end of the post-intervention year, two participants reported an unfavorable change-score of 1 and 2 for the PGIC.

### HEALTHCARE UTILIZATION

There was no statistically significant difference in healthcare utilization between the second intervention year and the following post-intervention year. However, the average number of COPD-related hospitalizations per patient per year doubled during the post-intervention year compared to the previous year when the patients were still participating in the intervention. The number of yearly COPD-related outpatient visits almost tripled during the year following the intervention (Table 6). Hence, the estimated COPD-related hospital costs also increased.

### MOTIVATIONAL FACTORS

Two themes regarding motivational factors for maintaining exercise were identified in the open-ended questionnaires collected at T3: (i) experiences of health improvements and (ii) regularity, feedback and affiliation.

#### EXPERIENCES OF HEALTH IMPROVEMENTS

Most participants described previous or current experiences of health improvements related to exercising as reasons why they continued to exercise:

- *I have certainly experienced that I benefit from physical activity and interval training* (P8).
- *I keep good health when I exercise and I recover faster after a flu. I get a lot of positive feedbacks from others. People who have not seen me for a while can hardly recognize me, and they say that I look healthy* (P1).

One participant stated that his motivation for exercise was *a wish for not getting worse* (P6).

#### REGULARITY, FEEDBACK AND AFFILIATION

Most of the participants expressed they missed the regularity and the feedback that the telerehabilitation intervention provided them during the pilot study:

- *I have missed the “pressure” to exercise weekly that came with the follow-up* (P3).

However, some participants did not miss the telerehabilitation follow-up:

- *I do not really miss the follow-up. However, I have not been diligent with my training (P4).*

The affiliation to the project and the group during the intervention seemed to have made an impression on the participants, and they *liked to be a part of the project* (P8) and after the intervention ended they *miss getting together to share experiences* (P2). Participants also highlighted *the training instruction and feedback* (P1), and *the conversations and being motivated by the physiotherapist* (P7) as something that they missed during the post-intervention period.

## DISCUSSION

Physical inactivity is associated with increased risk of all-cause mortality in COPD, and might also influence the clinical evolution of COPD (Van Remoortel et al., 2013; Waschki et al., 2011; Watz et al., 2014). Physical activity is reduced even from the early stages of the disease, in terms of both quantity and movement intensity (Van Remoortel et al., 2013; Watz et al., 2014). The American College of Sports Medicine recommends that adults engage in moderate physical activity for a total of ≥150 minutes per week (Garber et al., 2011). It is not known to what extent these recommendations apply to patients with COPD (Watz et al., 2014). Nevertheless, most patients with COPD do not meet these recommendations (Watz et al., 2014). In our study, the average time spent in MVPA bouts at the end of the post-intervention year was halved from that at the end of the intervention. This was still just above the recommendation of 150 minutes per week. However, analyzing the time spent in MVPA bouts individually, only two participants met the recommendations at the end of the post intervention year. The number of steps per day at the end of the telerehabilitation intervention was low and decreased even more and significantly at the end of the post-intervention year. The expected average value amongst free-living patients with COPD was estimated at 2237 steps per day (Tudor-Locke, Washington, & Hart, 2009). In our study, the average number of steps per day was just above this value at the end of the post-intervention year. However, we consider the difference between time points to be clinically important, as the MID after PR is estimated between 600 and 1100 steps per day (Demeyer et al., 2016). Further, there seems to be a discrepancy between the number of steps obtained and the time spent in MVPA bouts in our data. Thirty minutes spent in MVPA bouts would in healthy subjects translate to 7000–8000 steps per day (Tudor-Locke et al., 2011), whereas participants in our study had on average only 3806 ± 1596 steps from 49 ± 46 minutes in MVPA bouts (end of intervention). One reason for the discrepancy might be that accelerometers like the SenseWear activity monitor is worn on the upper arm unlike pedometers, which often are positioned at the hip and therefore gives more accurate measures of steps (Andersson, Janson, & Emtner, 2014). However, the SenseWear might be better in measuring MVPA due to its several heat-related sensors, which provide a good indication of the intensity of activity performed (Andersson et al., 2014; Cavalheri et al., 2011). Rollator use is known to influence the number of steps detected by the SenseWear (Andersson et al., 2014). Holding on to the handrails while walking on the treadmill might also influence the number of steps detected. The number of steps might have therefore been underestimated in this study. Nevertheless, the level of physical activity that the participants performed one-year post intervention seems not to be enough to maintain or achieve additional health benefits.

Maintenance of physical activity after PR is generally difficult (Spruit et al., 2013; Watz et al., 2014). Factors supporting maintenance of physical activity after PR from the patients’ view include reconciliation with the disease, professional support, adaption of intensity of the exercise program, feeling of mastery, social support from peers, and access to appropriate exercise facilities with regular supervision (Hellem et al., 2012). Most of the participants in our study stopped using the webpage for self-monitoring during the post-intervention year without any supervision provided by the physiotherapist via telerehabilitation. Adherence to training sessions also declined. This may have contributed to the significant decrease in physical activity levels compared to the previous telerehabilitation period. Only two participants continued using the webpage during the post-intervention year. They also had the highest adherence to telerehabilitation. These participants seemed to have effectively integrated exercise and self-management routines into their everyday life over the long term. Moreover, during the year following telerehabilitation, they started reporting more detailed and varied descriptions of their activities on the webpage, including other forms of physical activity besides treadmill training. There have been some, albeit few studies reporting telemedicine interventions for self-management support and exercise maintenance delivered without supervision or input from health personnel. Interventions seemed likely to be successful if they were individually tailored to fit the patients’ needs and skills, thus better integrated in everyday life and health care routines (Vassilev et al., 2015). It appears that the unsupervised version of the telerehabilitation intervention fit the needs of only two participants. The rest of the participants stopped adhering to the intervention as the professional support and regular supervision ceased.

Besides tailoring the intervention to fit the participants, development of relationships is recognized as a key component for successful telemedicine interventions (Vassilev et al., 2015). Results from the open-ended questionnaires on motivational factors for maintaining exercise during the post-intervention year support the importance of established relationships. These findings also confirm the results from focus groups conducted during the telerehabilitation intervention (Hoaas et al., 2016). Motivation for maintaining exercise was supported by experienced health benefits. In addition, some of the participants missed the regularity of feedback, motivational support and the specialized and individualized follow-up from a physiotherapist. Visibility of symptoms and exercise sessions through telemonitoring on the webpage and regular contact with the physiotherapist during the intervention period might have worked as a reinforcement or incentive as it encouraged accountability and a mild “pressure” to exercise (Vassilev et al., 2015). Therefore, increased attention seemed to be a key motivational factor for exercise maintenance. However, according to quotations from the open-ended questionnaires, this does not necessarily need to be provided by health personnel via telerehabilitation. Positive feedback and attention from other people might also be sufficient to continue exercising.

## LIMITATIONS

In our study, health status, health-related quality of life and other patient-related outcomes like anxiety and depression, self-efficacy, and subjective impression of change remained unchanged during the post-intervention year. However, it must be acknowledged that our small study may have been underpowered to detect changes in these outcomes. Quality of life is often maintained better than physical activity and exercise capacity in the long term after conventional pulmonary rehabilitation (Spruit et al., 2013). For the one study identified assessing long-term benefits of telerehabilitation for patients with COPD, improvements measured in quality of life during the intervention were not present 24 weeks later (Marquis et al., 2014).

In addition to the personal burden of having COPD, the disease poses a substantial economic burden on the public health system (García-Polo et al., 2012). In our study, there was no statistically significant change in COPD-related healthcare utilization between the time points. Participants might have been able to maintain their self-management routines during the post-intervention year after withdrawal of the supervision from the physiotherapist. However, the clear increase in the average number of hospitalizations and outpatient visits makes it difficult to be certain of this. COPD is a progressive disease. This might have influenced the tendency for more frequent hospitalization that we see in the average numbers. Since lung function was not measured at the end of the post-intervention year, we do not know whether participants had experienced a progression of the disease. A control group was not included for comparison. These are clearly limitations to the study. However, participants had reduced physical activity level and this is also known to influence the risk of hospitalization (Garcia-Aymerich et al., 2006; García-Polo et al., 2012; Watz et al., 2014).

Although this study is unique due to the long-term duration of the intervention and the additional reassessment one-year post intervention, generalization of results is hampered by the small sample size and uncontrolled nature of the study. Further, it is difficult to draw conclusions regarding which specific patients’ characteristics in terms of gender, residential status, disease severity, and BMI are related to maintenance of physical activity. Also, in the questionnaire regarding the use of the equipment, six participants answered they had used the treadmill and the pulse oximeter monthly or more often. This means that some participants might have exercised on the treadmill without registering the training sessions on the webpage, as this was voluntary during the post-intervention year. Adherence to training might therefore have been underestimated by the analysis of the logs from the webpage. Four participants also reported using the webpage monthly or more often. However, this is not reflected by the logs on the webpage. Self-reported data on patient questionnaires often overestimate objectively measured data. As a consequence, the logs on adherence to daily self-management registrations are more accurate.

## CONCLUSIONS

This paper examined whether physical activity and other outcomes obtained after participation in a two-year telerehabilitation intervention for COPD patients could be maintained over the following year without any supervision provided by a physiotherapist via telerehabilitation. We conclude that provision of equipment for self-management and home exercise might not be enough to maintain long-term effects on physical activity levels. Maintenance strategies including regular supervision, feedback or input by health professionals might better support long-term maintenance of physical activity and adherence. However, the cost-effectiveness of such interventions remains unknown.

## Figures and Tables

**Table 1:** Study Objectives and Time Points for Data Collection

Objectives	Measure	End of telerehabilitation, T2	End of post-intervention year, T3
Age	Year	X	
Lunge capacity	FEV1 (% of pred)	X	
Use of LTOT	Yes/no	X	
Body weight status	BMI	X	
Functional walking capacity	6 MWD	X	
Residential status	Living alone or not	X	
Physical activity levels	Activity monitor	X	X
Adherence to intervention	Logs on webpage during one year	X	X
Health status	CAT	X	X
Health-related quality of life	EQ-5D	X	X
Anxiety and depression	HADS	X	X
Self-efficacy	GSES	X	X
Subjective impression of change	PGIC	X	X
Healthcare utilization	COPD-related hospitalizations and outpatient visits	X	X
Motivational factors for maintaining exercise	Open-ended questionnaire		X

FEV_1_(% of pred)= Forced expiratory volume in 1 second, percentage. LTOT= Long-term oxygen treatment. BMI= Body Mass Index. 6MWD= 6 minute walk distance. CAT= the COPD Assessment Test. EQ-5D= the EQ-5D questionnaire. HADS= the Hospital Anxiety and Depression Scale. GSES= the General Self-Efficacy Scale. PGIC= the Patient Global Impression of Change

**Table 2:** Patients’ Characteristics at the End of the Telerehabilitation Intervention, T2

Variable	End of telerehabilitation, T2
Sex, males/females	5/4
Age, year	58.1 ± 6
FEV1, % of predicted	42.4 % ± 19.8
Use of LTOT, yes/no	4/5
BMI	25.7 ± 4.6
6MWD, meters	467.7 ± 106.7
Living alone, yes/no	5/4

FEV_1_, % of predicted= Forced expiratory volume in 1 second, percentage. LTOT= Long-term oxygen treatment. 6MWD= 6 minute walk distance.

**Table 3:** Physical Activity (Second Year of the Telerehabilitation vs. Post-intervention Year)

Physical activity	Intervention year 2 (n=7)	Post-intervention year (n=7)	Mean difference (95%CI)	P-value
Wear time, min/day	1342 ± 68	1329 ± 79	−13 (−81, 55)	0.653
Measured energy expenditure, kJ/day	9451 ± 2635	8270 ± 1846	−1181 (−1981, −381)	0.011
Steps, number/day	3806 ± 1596	2817 ± 1968	−989 (−1914, −66)	0.039
Awake sedentary time, min/day	654 ± 86	653 ± 124	−1 (−112, 109)	0.973
Total LIPA, min/day	187 ± 48	147 ± 67	−40 (−67, −14)	0.009
Total MVPA, min/day	88 ± 66	43 ± 34	−45 (−91, 1)	0.053
Time in MVPA bouts, min/day	49 ± 46	22 ± 27	−26 (−61, 8)	0.110

LIPA= Light physical activity. MVPA= Moderate-to-vigorous physical activity.

**Table 4:** Adherence (Second Year of the Telerehabilitation vs. Post-intervention Year)

Adherence	Intervention year 2 (n=9)	Post-intervention year (n=9)	Mean difference (95%CI)	P-value
Diary registrations per week	2.8 ± 2.2	1.1 ± 2.2	−1.7 (−2.8, −0.5)	0.010
Training sessions per week	1.3 ± 0.7	0.4 ± 1.0	−0.9 (−1.4, −0.3)	0.005
Diary registration % recommended	39.3 ± 31.1	15.6 ± 32.0	−23.8 (−40.0, −7.5)	0.010
Training sessions % recommended	42.9 ± 24.3	13.9 ± 31.7	−29.0 (−46.1, −11.9)	0.004

Recommendations for diary registrations and training sessions were 7 and 3 times/week, respectively.

**Table 5:** Patient-related Outcomes (Second Year of the Telerehabilitation vs. Post-intervention Year)

Patient-related outcomes	Intervention year 2 (n=9)	Post-intervention year (n=9)	Mean difference (95%CI)	P-value
CAT, total score	20.9 ± 6.8	19.1 ± 5.2	−1.8 (−6.3, 2.7)	0.388
EQ-5D, utility score	0.575 ± .215	0.715 ± .146	0.140 (0.014, 0.293)	0.070
EQ-5D, VAS scale	47 ± 24	51 ± 26	4 (−21, 29)	0.718
HADS, anxiety	6.67 ± 3.6	6.78 ± 3.1	0.11 (−1.90, 2.13)	0.902
HADS, depression	4.22 ± 2.3	4.78 ± 2.5	0.56 (−1.13, 2.24)	0.468
HADS, total score	10.89 ± 5.1	11.56 ± 3.6	0.67 (−2.73, 4.06)	0.663
GSES, total score	30.6 ± 4.9	30.0 ± 5.3	−0.6 (−2.8, 1,7)	0.584
PGIC score	5.4 ± 1.2	5 ± 2.1	−0.4 (−2.0, 1.1)	0.537

CAT= the COPD Assessment Test. EQ-5D= the EQ-5D questionnaire. HADS= the Hospital Anxiety and Depression Scale. GSES= the General Self-Efficacy Scale. PGIC= the Patient Global Impression of Change

**Table 6:** Healthcare Utilization (Second Year of the Telerehabilitation vs. Post-intervention Year)

Healthcare utilization	Intervention year 2 (n=9)	Post-intervention year (n=9)	Mean difference (95%CI)	P-value
COPD-related hospitalisations, number	0.44 ± 0.73	0.89 ± 1.27	0.45 (−0.11, 1.00)	0.104
COPD-related outpatient visits, number	1.22 ± 0.83	3.55 ± 7.00	2.33 (−3.12, 7.78)	0.352
COPD-related hospital costs, NOK	27,376 ± 45,661	54,816 ± 60,036	27,439 (−7,784, 62,663)	0.110
